# Pharmaceutical compounding of aflibercept in prefilled syringes does not affect structural integrity, stability or VEGF and Fc binding properties

**DOI:** 10.1038/s41598-018-20525-8

**Published:** 2018-02-01

**Authors:** Magne Sand Sivertsen, Øystein Kalsnes Jørstad, Algirdas Grevys, Stian Foss, Morten Carstens Moe, Jan Terje Andersen

**Affiliations:** 1 Department of Ophthalmology, Oslo University Hospital and University of Oslo, Oslo, Norway; 2 Centre for Immune Regulation (CIR) and Department of Immunology, Oslo University Hospital and University of Oslo, Oslo, Norway; 3 Department of Biosciences, University of Oslo, Oslo, Norway

## Abstract

Macular edema due to neovascular age-related macular degeneration, diabetes or retinal vein occlusion can cause central vision loss. Intravitreal treatment with antibody-based biopharmaceutical compounds designed to neutralize vascular endothelial growth factor (VEGF) has proven to be an efficient strategy to ameliorate macular edema and restore visual acuity. At the same time, the use of anti-VEGF drugs places an economic burden on the health care system; the drugs are expensive, and repeated injections are usually required to maintain the therapeutic effect. Thus, there is an unmet need for more cost-effective procedures. We here describe how the most recently approved anti-VEGF drug, aflibercept, can be compounded into prefilled sterile syringes and stored for up to 4 weeks without compromising its quality, stability or functional properties, including VEGF and neonatal Fc receptor (FcRn) binding. The novel compounding method for repackaging of aflibercept in sterile plastic syringes can greatly reduce both cost and time spent per patient in the injection room.

## Introduction

Over the last decade, antibody-based biopharmaceutical compounds designed to target vascular endothelial growth factor (VEGF) have revolutionized treatment and prognosis for several retinal diseases, including wet age-related macular degeneration^1,2^, diabetic macular edema^3^ and macular edema secondary to retinal vein occlusion^4^. The three most commonly used anti-VEGF drugs are aflibercept (Eylea®, Bayer Healthcare), ranibizumab (Lucentis®, Genentech) and bevacizumab (Avastin®, Genentech). Aflibercept and ranibizumab are approved for ophthalmological use by national and multinational drug agencies, while bevacizumab is an off-label alternative whose efficacy and safety are well established through extensive clinical research^5–8^.

These costly drugs are administered as intravitreal injections, and regular retreatment is usually necessary to achieve and maintain therapeutic effect. Their introduction as the standard of care in several prevalent conditions has thus led to a greatly increased workload for ophthalmological clinics, and has placed a heavy economic burden on health care systems. Intravitreal injections also involve a risk of infectious endophthalmitis. The incidence of this rare but sight-threatening complication depends on the injection procedure used^9,10^. In this context, a standardized, quality-controlled, time- and cost- efficient injection practice is paramount, and any means of improving either efficiency or safety is invaluable.

Aflibercept is the anti-VEGF drug that was most recently approved for treatment of ocular diseases. It is a recombinant fragment crystallizable (Fc) fusion protein consisting of parts of the extracellular domains of human VEGF receptors 1 and 2 that are genetically fused to the Fc portion of human IgG1. Compared to other anti-VEGF drugs aflibercept has a higher binding affinity for VEGF^11^, longer intravitreal half-life^12^ as well as the capacity to antagonize growth factors other than VEGF^12^.

Compounded preparations of bevacizumab are commercially available in many countries; pharmacies split vials into injection-ready syringes that are sold to ophthalmological clinics. Although aflibercept is supplied in considerably smaller vial volumes than bevacizumab, a compounding procedure with splitting of each aflibercept vial into several syringes would have a large impact on treatment costs. In addition to reducing costs, the production of prefilled syringes in a pharmacy rather than in the injection room can potentially lower the risk of microbial contamination^13^. It will also be time-saving for the clinician^14^. However, compounding and storage of antibody-based drugs in plastic syringes diverges from the administration approved by manufacturers and drug authorities. Thus, any compounding procedure must be carefully controlled to guarantee its security and quality.

In this study, we describe a novel pharmacy compounding method for repackaging of aflibercept into sterile plastic syringes. A thorough investigation shows that aflibercept fully maintains its structural stability, VEGF and Fc binding properties after up to 28 days of storage.

## Results

To investigate whether aflibercept can be compounded and stored in sterile plastic syringes without affecting protein integrity or VEGF and Fc binding properties, commercially obtained aflibercept was drawn into insulin plastic syringes in an isolator unit and transfer chamber following aseptic production procedures. The prefilled syringes were stored at 4 °C in the dark for 0, 7 or 28 days (D0, D7 and D28). The aliquots were then immediately analyzed using an array of methods to measure the quality, stability and binding properties of aflibercept.

### Protein quality and stability

**Figure 1:**
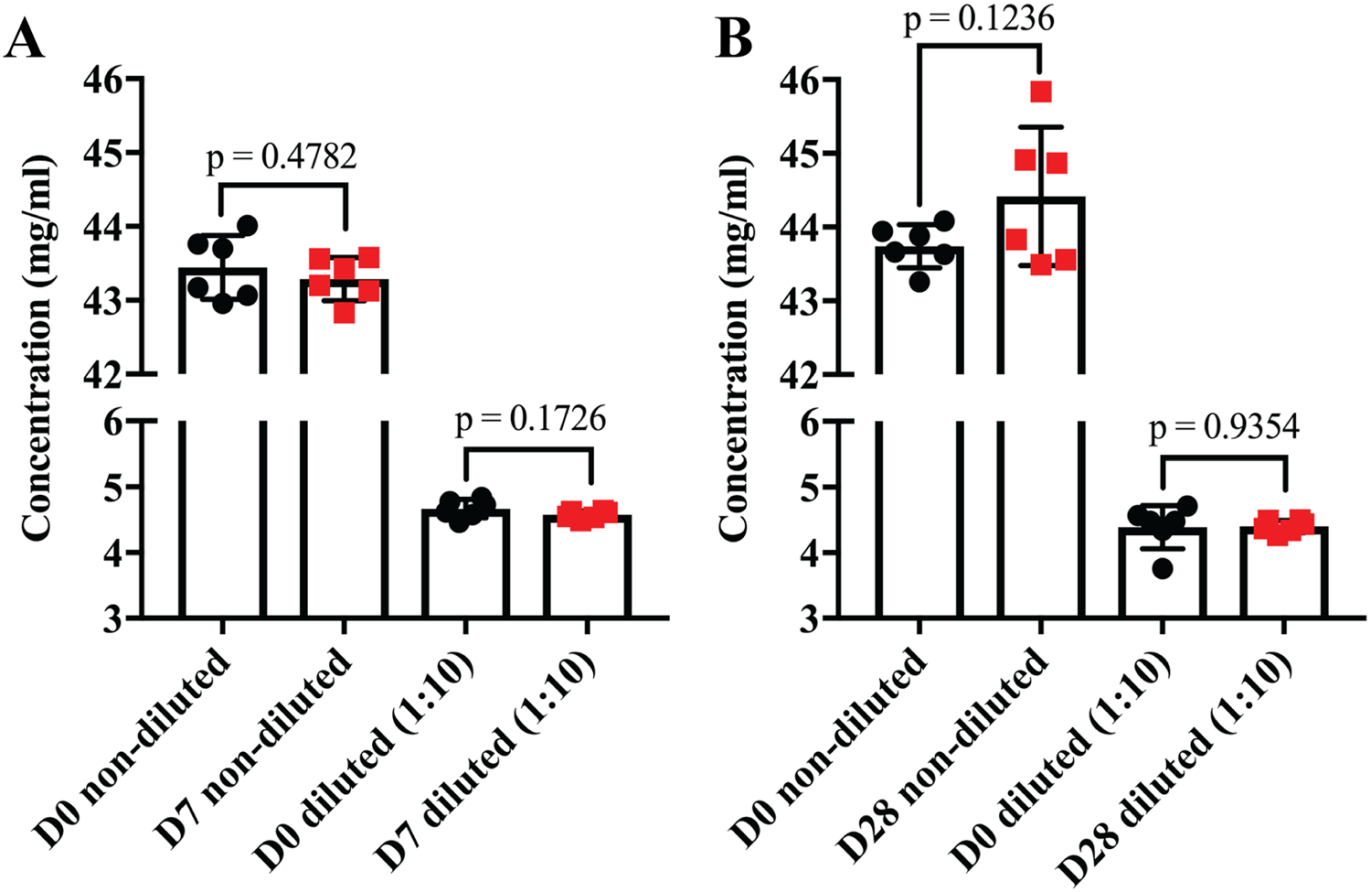
Concentration of aflibercept stored in prefilled plastic syringes. Concentration measurements of non-diluted and diluted (1:10) aflibercept samples collected at (**A**) D0 and D7, and (**B**) D0 and D28. Data are presented as mean ± S.D. (n = 6 for each comparison). Unpaired Student’s t-test was used for statistical calculations.

We initially determined the concentration of aflibercept in the plastic syringes. Aflibercept was collected from the syringes and both undiluted and diluted (1:10) samples were measured using a DeNovix DS-11+ Spectrophotometer. There were no statistically significant differences in concentrations between samples collected at D0, D7 and D28 (Fig. 1A,B). Thus, the storage in syringes did not result in any loss of protein over 4 weeks.

**Figure 2:**
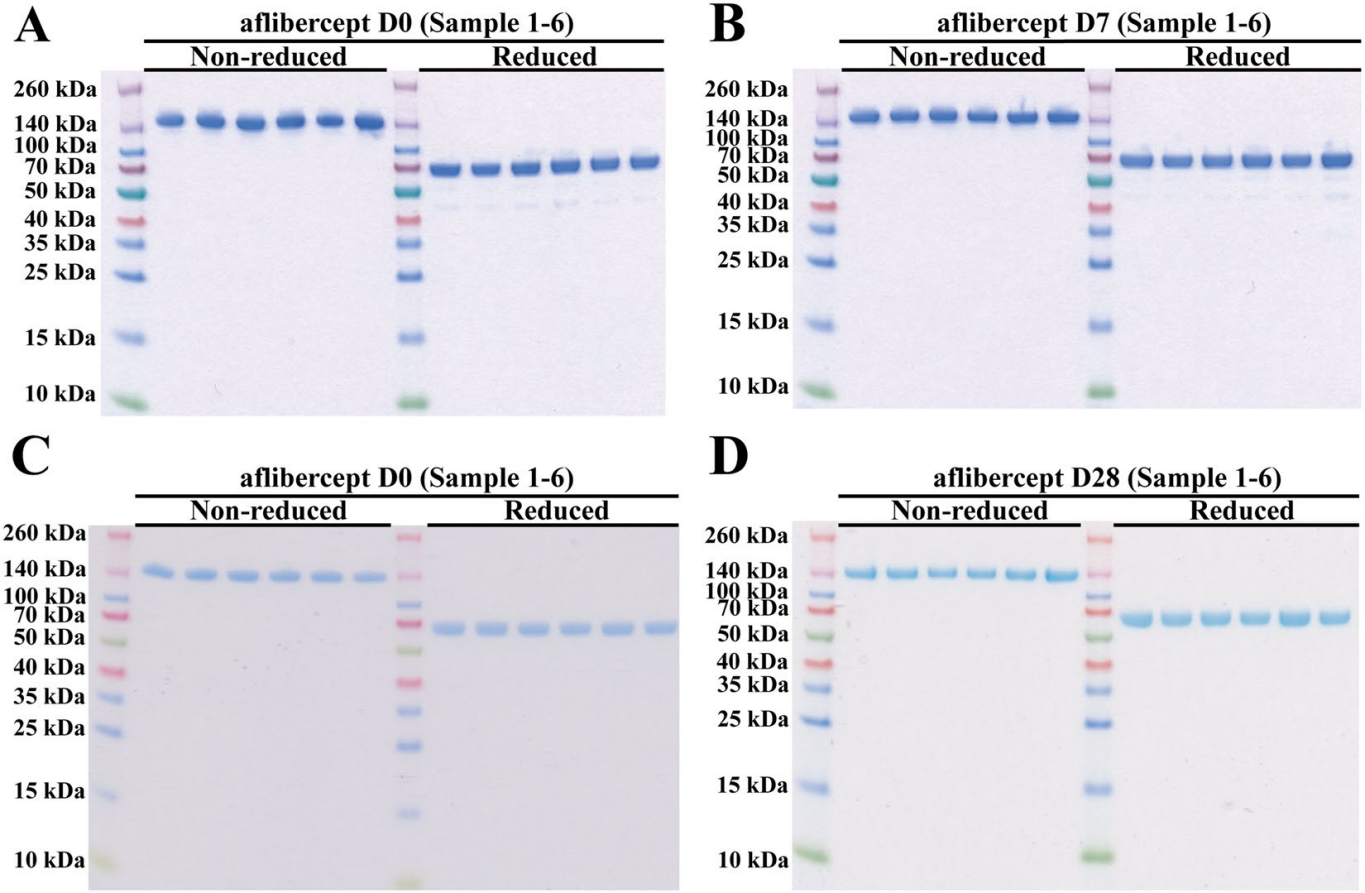
SDS-PAGE analysis for aflibercept. Non-reducing and reducing SDS-PAGE analysis of aflibercept samples from (**A**,**B**) D0 and D7, and (**C**,**D**) D0 and D28. The gels in (**A**,**B**) and (**C**,**D**) were processed in parallel. The images have not been cropped.

We then analyzed the structural integrity of aflibercept by adding equal amounts on SDS-PAGE gels under non-reducing and reducing conditions (Fig. 2A–D). Under non-reducing conditions all fractions migrated as major bands corresponding to the aflibercept homodimer with a molecular weight of ~150 kDa. Under reducing conditions, where the disulfide bonds of the IgG1 Fc hinge region are disrupted, aflibercept migrated, as expected, as a major band of ~70 kDa. Notably, under reducing conditions we observed two additional minor bands with molecular weights of roughly 55–45 kDa. These bands were found for all storage durations including D0.

**Figure 3:**
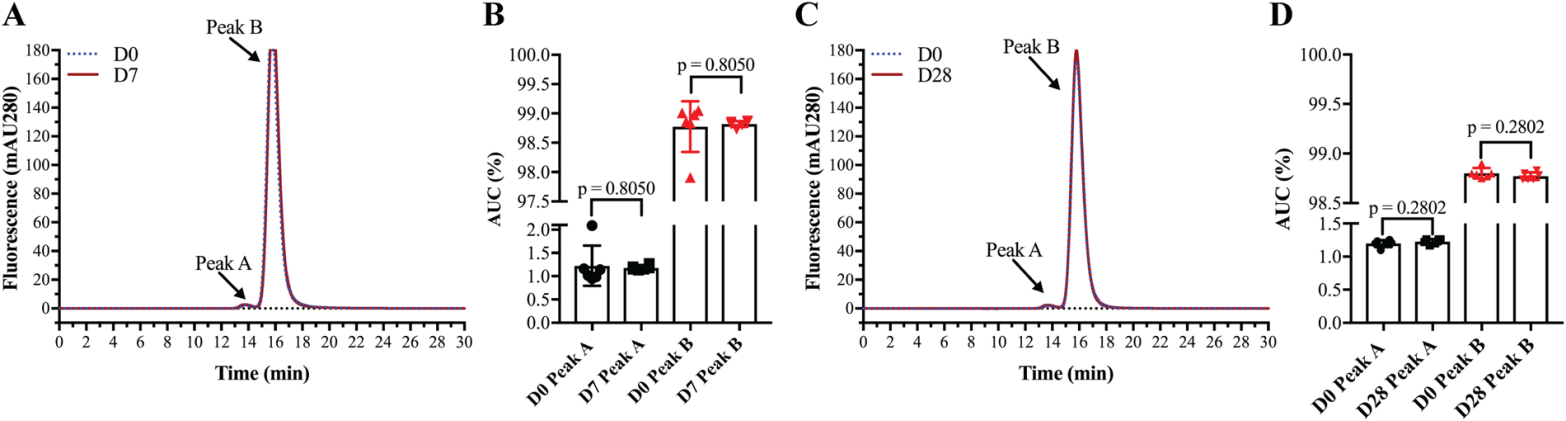
SEC analysis of aflibercept. (**A**) SEC elution profiles of aflibercept samples from D0 and D7. (**B**) Comparison of AUC (%) of peak A and peak B for aflibercept samples from D0 and D7. (**C**) SEC elution profiles of aflibercept samples from D0 and D28. (**D**) Comparison of AUC (%) of peak A and peak B for aflibercept samples from D0 and D28. The data are presented as mean ± S.D. n = 6 for each comparison. Unpaired Student’s t-test was used for statistical calculations.

**Figure 4:**
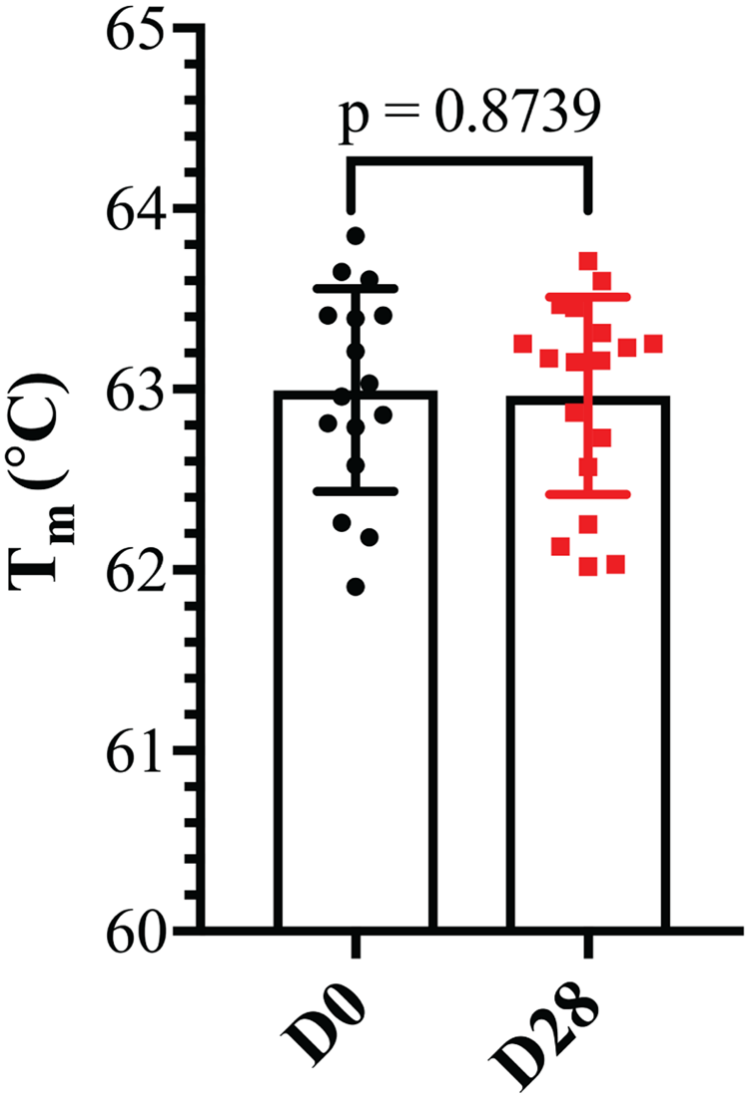
Thermal stability of aflibercept. T_m_ values for aflibercept at D0 and D28. The data are presented as mean ± S.D. From one independent experiment performed in triplicates (n = 12). Unpaired Student’s t-test was used for statistical calculations.

The aflibercept samples were also analyzed with size exclusion chromatography (SEC). All fractions showed identical elution profiles with a major peak (peak A) corresponding to homodimeric aflibercept. A minor peak (peak B) corresponding to a dimeric form of homodimeric aflibercept was also seen in all samples (D0, D7 and D28, Fig. 3A,C). Calculation of the area under the curve (AUC) showed no statistically significant differences between the D0, D7 (p = 0.805) and D28 samples (p = 0.280) (Fig. 3B,D, n = 6 for each comparison). Finally, thermal stability was measured for D0 and D28 samples by differential scanning fluorimetry (DSF). Again, no statistically significant differences were found, as all samples reached 50% unfolding (Tm value) at 62.9 °C (p = 0.874, Fig. 4).

Thus, storage of aflibercept in sterile prefilled syringes did not affect thermal stability or cause degradation or formation of covalent or non-covalent aggregates.

### Antigen and Fc binding properties

Preserving the functional integrity of therapeutic antibodies during manufacture and storage represents a major challenge. Aflibercept has two inherent functional properties; it binds VEGF via VEGF receptor subunits fused to the N-terminal end of each of the Fc heavy chains, and to Fc receptors via constant Fc domains derived from human IgG1. One of the most important Fc receptors is the neonatal Fc receptor (FcRn), which is responsible for the 3-week serum half-life of IgG^15,16^. A finding relevant to storage stability is that oxidation of methionine residues on the Fc part of IgG1 causes reduced binding to FcRn, consequently shortening the serum half-life^17,18^. Presumably, this would also affect the dynamics of intravitreally administered drugs containing an Fc portion (aflibercept and bevacizumab), as FcRn has been shown to play a central role in removing intravitreally administered IgG via the blood-retinal barrier^19^.

**Figure 5:**
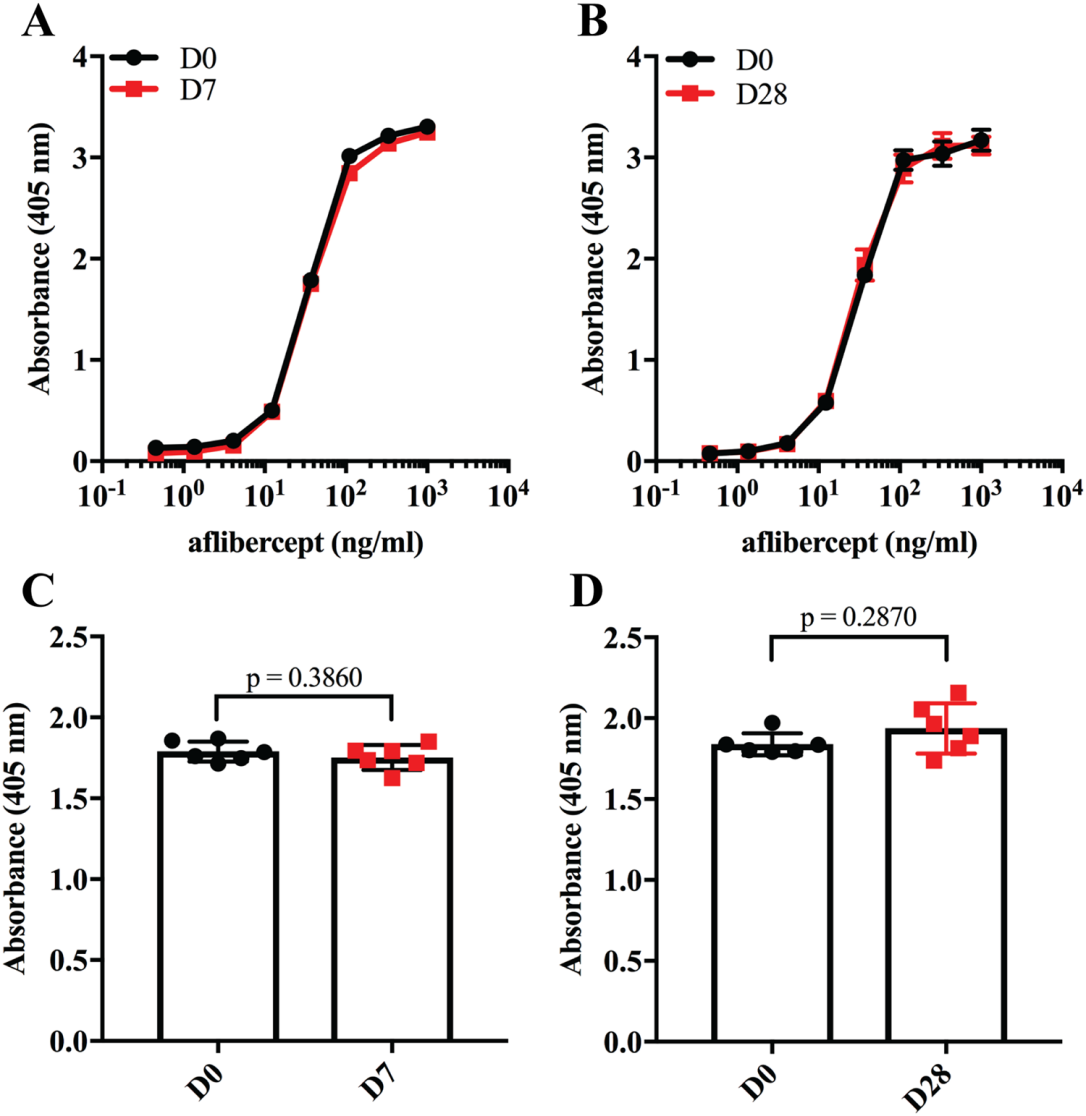
VEGF binding properties of aflibercept. Binding of titrated amounts (1000.0–0.5 ng/ml) of aflibercept to VEGF_165_ at (**A**) D0 and D7, and (**B**) D0 and D28 in ELISA. (**C**) Comparison of aflibercept (37 nM) binding to VEGF_165_ at D0 and D7, and (**D**) at D0 and D28. Unpaired Student’s t-test was used for statistical calculations.

**Figure 6:**
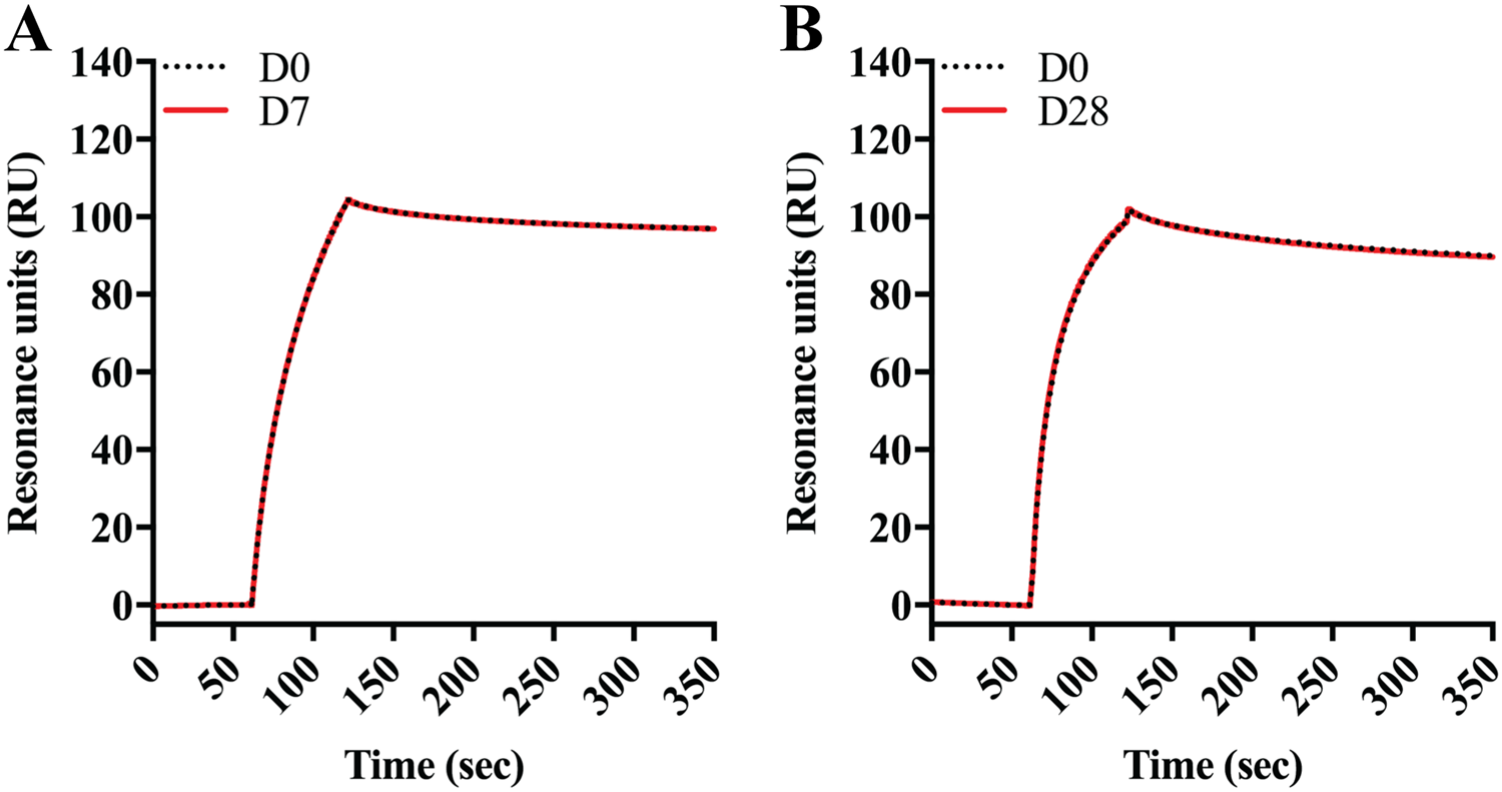
Binding of aflibercept to VEGF using SPR. Representative sensorgrams showing overlays of the binding profiles of aflibercept (100 nM) samples from (**A**) D0 and D7, and (**B**) from D0 and D28 to immobilized human VEGF_165_ (~300 RU). Each curve represents the average of six individual injections. The binding responses (RU) have been reference subtracted and normalized for clarity.

To investigate how the aflibercept compounded and stored in syringes bound human VEGF, ELISA was performed; the plates were coated with constant amounts of VEGF before titrated amounts of aflibercept were added. Captured aflibercept was detected using a polyclonal anti-human IgG Fc antibody. The binding profiles obtained showed that the VEGF binding integrity was visually indistinguishable for D7 and D28 samples compared to D0 samples (Fig. 5A,B). Statistical analysis of values derived from the exponential phase of the curves showed no significant differences in VEGF binding capacity (D7: p = 0.386, D28: p = 0.287, Fig. 5C,D). This could also be shown using surface plasmon resonance (SPR) where VEGF was immobilized and equal amounts of aflibercept were injected. The resulting sensorgrams showed completely overlapping binding profiles (Fig. 6A,B), which confirmed that the VEGF binding properties were unaffected by storage.

**Figure 7:**
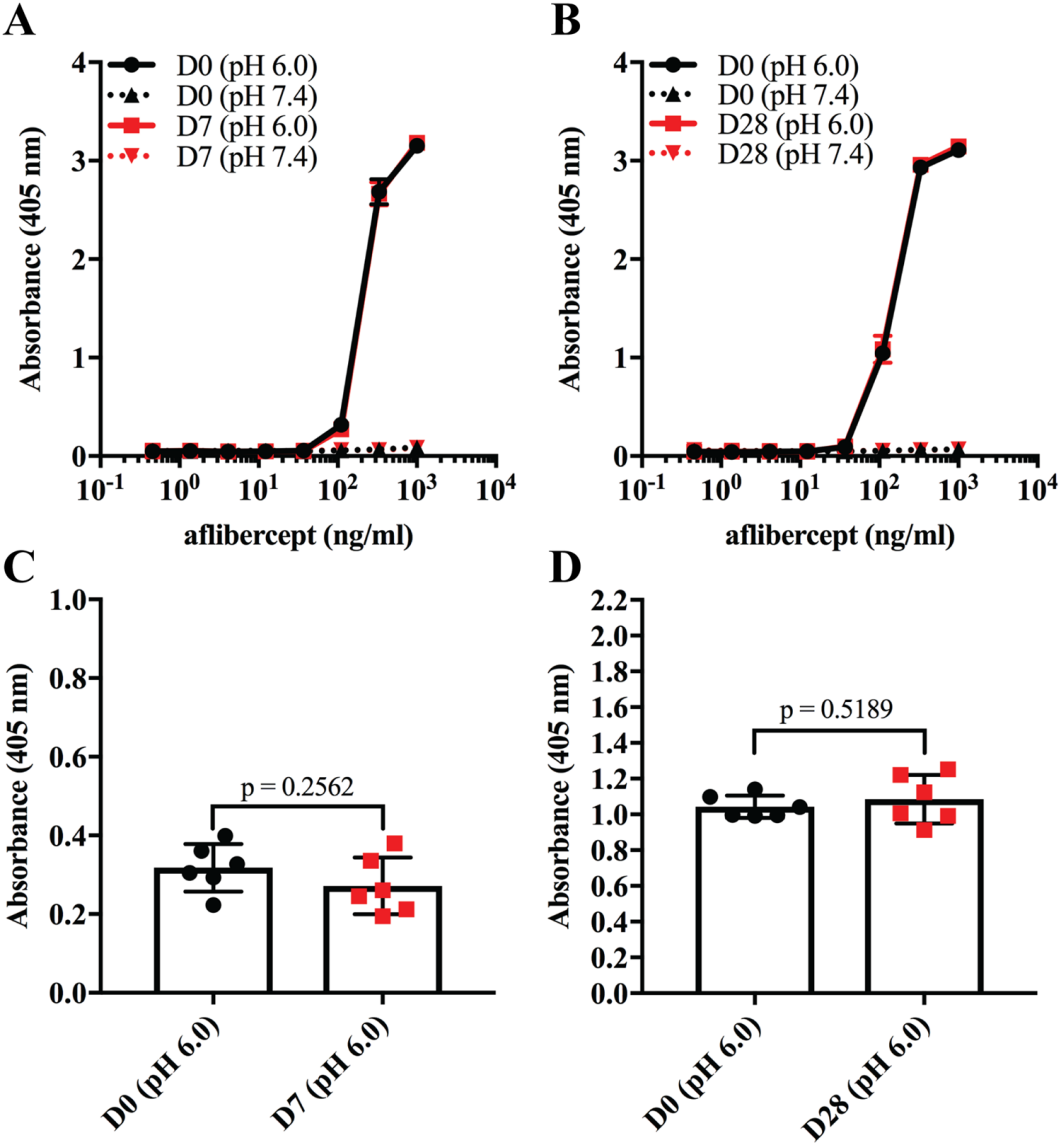
FcRn binding properties of aflibercept. Binding of titrated amounts (1000.0–0.5 ng/ml) aflibercept from (**A**) D0 and D7, and (**B**) D0 and D28 to GST-tagged human FcRn at pH 6.0 and 7.4 in ELISA. (**C**) Comparison of aflibercept (111 nM) binding to human FcRn at D0 and D7, and (**D**) D0 and D28 at pH 6.0. The data represent mean ± S.D of duplicates from one independent experiment (n = 6). Unpaired Student’s t-test was used for statistical calculations.

To evaluate the functional integrity of the Fc part of aflibercept, binding to human FcRn was measured by ELISA. Since FcRn binds IgG in a strictly pH dependent manner, i.e. binding at acidic pH and no binding or release at pH 7.4^15,16^, the assay was performed at both pH 7.4 and pH 6.0. Specifically, titrated amounts of aflibercept were captured on VEGF followed by adding of recombinant GST-tagged human FcRn and detection using an anti-GST antibody (Fig. 7A,B). The results showed that human FcRn bound aflibercept stored for 7 or 28 days equally well and in the same pH dependent manner as freshly drawn aflibercept (Fig. 7A,B), without statistically significant differences (Fig. 7C,D).

Thus, up to 28 days storage of alifbercept in prefilled syringes did not affect either of the two functional binding properties of aflibercept, neither binding to VEGF nor pH dependent binding to human FcRn.

## Discussion

The practice of pharmacy compounding of anti-VEGF antibody-based drugs essentially dates back to their introduction. Compounding has been most widespread for bevacizumab, and a large body of research exists demonstrating functional stability of bevacizumab for up to 6 months of storage in pharmacy-produced syringes^20–26^. Considering ranibizumab, there are two reports with conflicting results; one reports reduced VEGF binding activity after 1 week^26^, while the other reports unaltered activity after 4 weeks^27^.

We describe a novel method for compounding of aflibercept into sterile syringes prepared for intravitreal injection. When stored in the dark at 4 °C a range of assays show that the molecular integrity, stability and functional properties of aflibercept remain unaffected for up to 4 weeks. The present paper is the first to present such a thorough investigation. Adding to the evidence, a recent paper describes unaltered activity of aflibercept using an ELISA set-up where binding to placental growth factor instead of VEGF was measured and different syringes were used^27^. The authors conclude that aflibercept can be stored in plastic syringes for up to 4 weeks before use.

Aflibercept is also approved for treatment of metastatic colorectal cancer (Zaltrap, Sanofi-Aventis and Regeneron). The so-called ziv-aflibercept, which is produced by a different procedure, has higher osmolarity and lower drug concentration than aflibercept^28^. Regardless, there are many reports on intravitreal use of ziv-aflibercept^29–32^. It has been shown that ziv-aflibercept maintains some of its ability to bind VEGF after 4 weeks of storage in polycarbonate syringes^28^. However, a significant decline in affinity (61% reduction after 4 weeks of storage) suggests that the activity of ziv-aflibercept is negatively affected by the storage. This discrepancy from our findings can most likely be ascribed to the differences in production and formulation of the two aflibercept drugs.

In the current study, aflibercept from freshly drawn, injection-ready syringes were used as the reference (D0), and not aflibercept from the original vial. The rationale behind this choice was that the drug will always have to be withdrawn into a syringe before intravitreal injection. In a previous paper assessing the stability of bevacizumab in syringes prepared by 11 different compounding pharmacies, bevacizumab from the original vial was used as reference^25^. Using drug from the original vial as reference introduces a source of error, as protein stability and integrity may be affected simply by the withdrawal into a syringe, while the question of interest is whether it is affected by storage.

We chose four weeks storage duration to address how aflibercept was affected over time and to facilitate comparison with previous studies where the same timeframe has been used^20–24,26–28^. In a Norwegian context one week is the more relevant storage time, as this is the maximum shelf life allowed in Norway when an aseptic production procedure without renewed sterilization is employed. Our data demonstrate that fully active aflibercept ready for intravitreal injection can be guaranteed after one week of storage.

Both the aflibercept and bevacizumab molecules include an Fc portion derived from human IgG1. The Fc portion has been shown to be important for clearance of intravitreally injected IgG through interaction with FcRn^19^. In this study, we show that human FcRn binds aflibercept in a strictly pH dependent manner and that there is no differences in binding activity between D28 and D7 samples compared to D0 controls. This implies that intra-ocular transport kinetics are unaltered and thus should not interfere with the clinical efficacy. This is an aspect of bevacizumab and aflibercept function that has not been investigated in previous stability studies.

Despite being in line with the drug agency-approved administration, preparing syringes in the operating room involve a risk of bacterial contamination. The aseptic production described in this paper most likely reduces the risk of contamination and thereby lowers the risk of bacterial endophthalmitis^13^. This issue was not investigated in the present paper, as the sample size needed to obtain statistical power for this presumably very rare occurrence would have been prohibitive. Previous studies on bevacizumab, however, show no bacterial contamination of repackaged syringes^24,25^, but carry the same statistical caveat. In our clinic, we have used pharmacy-prepared aflibercept as described in this paper since March 2016 (until September 2017 practicing a shelf-life of 24 hours), and performed more than 10.000 intravitreal injections, so far without any cases of endophthalmitis. Although not enough to conclude statistically due to the low overall incidence of bacterial endophthalmitis, this indicates that our practice of pharmacy compounding is at least as safe as preparation of syringes in the operating room.

Aside from an apparent improvement in safety and decreased time spent per patient in the operating room^14^, the main benefit from pharmacy compounding is the reduced health care expenses. In 2016, our eye department performed around 10 000 intravitreal injections with aflibercept. Pharmacy compounding at our clinic alone thus saved an annual 44 million NOK (5.6 million USD) for the Norwegian public health care system.

In conclusion, aflibercept compounded into injection-ready syringes can be stored for 4 weeks without affecting neither VEGF nor FcRn binding properties and thus should retain unaltered clinical efficacy.

## Materials and Methods

### Repackaging process

Aflibercept was acquired commercially (Eylea®). Prefilled injection syringes intended for intravitreal injection were produced under standard conditions at the hospital pharmacy. The syringes were filled aseptically in an isolator unit with a class A production chamber and class B transfer chambers (classifications according to EU GMP^33^ and ISO 14644-1^34^). The production followed validated aseptic production procedures^33^, which involves closed systems, sterile single-use equipment and certified personnel. The contents of two vials of aflibercept (~0.22 ml/vial) were drawn into one syringe (Injekt-F, 1 ml, Braun). From the tip of this syringe, ~0.055 ml was drawn into each of six insulin plastic syringes (BD Micro-Fine Plus, 0.5 ml, 30 G, 8 mm, Becton, Dickson and Co.). The syringes were then capped, packed separately in sterile transparent plastic bags (Intervoid Steril, 150 × 220 ml, Coveris) and eventually visually inspected and labelled outside the isolator. The finished syringes were stored under dark conditions at 4 °C for 0, 7 or 28 days (D0, D7 and D28). The D7 and D28 experiments were performed on separate days, and were compared to separate sets of D0 samples. Thus, the D0 sample size was 12 while the D7 and D28 sample size was 6.

### Concentration measurements

Aflibercept samples were transferred from prefilled syringes to sterile Eppendorf tubes (Eppendorf) and incubated on ice while being protected from direct light. Each sample was diluted 1:10 in sterile phosphate buffered saline (PBS) (Sigma-Aldrich). The concentrations of both undiluted and diluted samples were determined using a DeNovix DS-11+ Spectrophotometer (Saveene Werner) following the manufacturer instructions. Two independent measurements were done for each sample and the average value calculated.

### SDS-PAGE analysis

Aliquots of 1 µg of aflibercept collected from prefilled syringes were mixed with distilled water and LDS sample buffer (Novex) with or without DL-dithiothreitol solution (Sigma-Aldrich). Samples containing DL-dithiothreitol were incubated for 5 min. at 95 °C before being applied onto 12% Bis-Tris Plus gel (Invitrogen) and run at 200 V for 22 min. Spectra™ Multicolor Broad Range Protein Ladder (ThermoFisher) was used for molecular size comparisons, and the protein bands were visualized by Coomassie staining (Bio-Rad).

### Size exclusion chromatography (SEC)

SEC was performed using an Avant-25 ÄKTA instrument (GE Healthcare) and Superdex 200 Increase 10/300 GL column (GE Healthcare). Aliquots of aflibercept collected from prefilled syringes were diluted in PBS (8 mg/ml) and 77 µl of each sample was injected using an auto-sampler (Spark). The sample compartment was kept at 4 °C while the analysis temperature was 25 °C.

### Anti-human Fc ELISA

96-well ELISA plates (Corning-Costar) were coated with 0.2 µg/ml human VEGF_165_ (Sigma-Aldrich) diluted in PBS (Sigma-Aldrich). After overnight incubation at 4 °C, plates were blocked with PBS containing 4% skimmed milk (S) (Sigma-Aldrich) for 1 hour at room temperature and washed 4 times with PBS containing 0.05% Tween-20 (T) (Sigma-Aldrich). Serial dilutions of collected aflibercept samples (1000.0–0.5 ng/ml) in PBS/S/T were added to the plates followed by incubation at room temperature for 1.5 hours. Plates were then washed as described above, before an alkaline phosphatase-conjugated goat polyclonal antibody directed towards human IgG Fc (Sigma-Aldrich), diluted (1:5000) in PBS/S/T, was added and incubated at room temperature for 1.5 hours. The plates were washed as above before 100 µl of the p-nitrophenylphospate substrate (Sigma-Aldrich), diluted to 10 µg/ml in diethanolamine buffer, was added. The absorbance was measured at 405 nm using a Sunrise spectrophotometer (TECAN).

### FcRn binding ELISA

96-well ELISA plates (Costar) were coated with 0.5 µg/ml human VEGF_165_ (Sigma-Aldrich) and blocked before serial dilutions of collected aflibercept samples were added as above. Plates were washed with PBS/T (pH 7.4) or 67 mM phosphate buffer (pH 5.5), before 1.0 µg/ml of GST-tagged receptor FcRn^35^, diluted in pH 7.4 or pH 5.5 buffer, was added and incubated at room temperature for 1.5 hours. The plates were washed as above before a horseradish peroxidase-conjugated goat polyclonal antibody directed towards GST (Rockland Immunochemicals Inc), diluted (1:8000) in pH 7.4 or pH 5.5 buffer, was added and incubated at room temperature for 1.5 hours. After washing as above, bound receptor was detected using the tetramethylbenzidine substrate (Calbiochem) and the reaction was stopped by adding 50 µl 1 M HCl. The absorbance was measured at 450 nm using a Sunrise spectrophotometer (TECAN).

### Surface plasmon resonance (SPR)

SPR was performed using a Biacore T200 instrument (GE Healthcare) with CM5 sensor chips coupled with human VEGF_165_ (Sigma-Aldrich) (~300 resonance units (RU)) using amine-coupling chemistry as described by the manufacturer. Briefly, the coupling was performed by injecting 5 μg/ml of VEGF diluted in 10 mM sodium acetate, pH 4.5 (GE Healthcare) using the amine-coupling kit (GE Healthcare). HBS-EP pH 7.4 buffer (0.01 M HEPES, 0.15 NaCl, 3 mM EDTA 0.005% surfactant P20) was used as both dilution and running buffer. Binding analysis was performed by injecting 100 nM of each aflibercept sample over the immobilized surface using a flow rate of 50 µl/min and 10 mM NaOH was used to regenerate the CM5 surface. The sample compartment was kept at 4 °C while the binding analysis was performed at 25 °C. The binding data were zero adjusted and the maximum binding responses of the individual injections were normalized using the BIAevaluation software version 4.1.

### Differential scanning fluorimetry (DSF)

Thermal stability of aflibercept was determined by DSF using a Lightcycler RT-PCR machine (Roche). SYPO Orange (Sigma-Aldrich) was used at a 1:500 dilution and protein concentration at 0.1 mg/ml in a final volume of 25 µl. All samples were run in triplicates in 96 well Lightcycler 480 Mulltiwell Plate (Roche). The peak of excitation and emission of SYPO Orange were 490 nm and 580 nm, respectively, and the 450 nm excitation and 568 nm emission filters on the RT-PCR machine were used. The RT-PCR machine was programmed to raise the temperature from 20 °C to 95 °C after a stabilization period of 10 min at 20 °C. Two measurements at each temperature were recorded, and data collected every 0.5 °C. Data transformation and analysis were performed using the DSF analysis protocol^36^.

### Statistical analysis

Figures and statistical analyses were performed using GraphPad Prism 7 for Windows (Version 7.02; GraphPad Software Inc.) and Microsoft Excel 2010 (Microsoft). P-values lower than 0.05 were considered statistically significant.

### Data availability

The datasets generated during and/or analyzed during the current study are available from the corresponding author on reasonable request.
